# Functional Re-organization of Cortical Networks of Senior Citizens After a 24-Week Traditional Dance Program

**DOI:** 10.3389/fnagi.2018.00422

**Published:** 2018-12-21

**Authors:** Vasiliki I. Zilidou, Christos A. Frantzidis, Evangelia D. Romanopoulou, Evangelos Paraskevopoulos, Styliani Douka, Panagiotis D. Bamidis

**Affiliations:** ^1^Laboratory of Medical Physics, Medical School, Aristotle University of Thessaloniki, Thessaloniki, Greece; ^2^Department of Physical Activity and Recreation, School of Physical Education and Sport Science, Aristotle University of Thessaloniki, Thessaloniki, Greece

**Keywords:** dancing, electroencephalography, neuroplasticity, dementia, active aging, functional connectivity, neurodegeneration, brain networks

## Abstract

Neuroscience is developing rapidly by providing a variety of modern tools for analyzing the functional interactions of the brain and detection of pathological deviations due to neurodegeneration. The present study argues that the induction of neuroplasticity of the mature human brain leads to the prevention of dementia. Promising solution seems to be the dance programs because they combine cognitive and physical activity in a pleasant way. So, we investigated whether the traditional Greek dances can improve the cognitive, physical and functional status of the elderly always aiming at promoting active and healthy aging. Forty-four participants were randomly assigned equally to the training group and an active control group. The duration of the program was 6 months. Also, the participants were evaluated for their physical status and through an electroencephalographic (EEG) examination at rest (eyes-closed condition). The EEG testing was performed 1–14 days before (pre) and after (post) the training. Cortical network analysis was applied by modeling the cortex through a generic anatomical model of 20,000 fixed dipoles. These were grouped into 512 cortical regions of interest (ROIs). High quality, artifact-free data resulting from an elaborate pre-processing pipeline were segmented into multiple, 30 s of continuous epochs. Then, functional connectivity among those ROIs was performed for each epoch through the relative wavelet entropy (RWE). Synchronization matrices were computed and then thresholded in order to provide binary, directed cortical networks of various density ranges. The results showed that the dance training improved optimal network performance as estimated by the small-world property. Further analysis demonstrated that there were also local network changes resulting in better information flow and functional re-organization of the network nodes. These results indicate the application of the dance training as a possible non-pharmacological intervention for promoting mental and physical well-being of senior citizens. Our results were also compared with a combination of computerized cognitive and physical training, which has already been demonstrated to induce neuroplasticity (LLM Care).

## Introduction

As the number of elderly people worldwide is constantly increasing, important changes regarding the mental, physical and emotional condition occurred. These changes refer to the brain structure, as well as to various functions related to aging. The alterations of the structure and function of the brain associated closely with changes in cognitive function. The most important cognitive functions affected by aging are considered to be memory, attention and movement that may lead to dementia or even Alzheimer’s disease.

Different parts of the brain, frontal cortex and hippocampus, were mostly affected by aging leading to the reduction of blood flow in the vessels by causing changes in amyloid plaques. High level of education, healthy lifestyle and regular exercise seem to be essential factors contributing on keeping older adults healthy, preventing diseases and reducing associated complications (Erickson et al., 2011). In recent decades, studies show that regular physical activity and exercise could mediate the negative effects of aging on both body and mind (Billis et al., 2010). The growing proportion of elderly people demonstrates the need of maintaining the independent living, the social integration and the active life for a longer period.

Neuroscience research has provided insights into the brain functions and behaviors with the main focus on the Mild Cognitive Impairment (MCI) transition by contributing to the development of neurodegenerative diseases. Therefore, a plethora of non-pharmacological interventions have been developed. During the last decades there is a steady interest on the investigation of the aging negative effects that can be eliminated through the physical and/or cognitive training. These training procedures aim to induce the neuroplasticity of the mature human brain (Bamidis et al., 2014). The brain is adapted to the new environment (brain and cognitive alterations) by enhancing existing or creating new connections. It is reported that aerobic exercise induces neurogenesis of hippocampus (Erickson et al., 2011) and physical activity is associated with the reduction of risk factors that may accelerate neurodegeneration (Cotman et al., 2007). Research findings show that physical activity is a promising non-pharmaceutical intervention to prevent cognitive impairment associated with age and neurodegenerative diseases (Bherer et al., 2013). In another study, the computerized physical training presented positive effects in the physical and cognitive capacity as well as the participants’ quality of life (Zilidou et al., 2016).

Long lasting memories (LLM) is developed as an integrated platform of existing technological achievements in the field of computer-aided physical/cognitive training and ambient assisted living (AAL) solutions for elderly people and vulnerable groups (Romanopoulou et al., 2015). LLM offers an opportunity to improve cognitive and physical condition, being able to continue feeling an active part of society. The core of the LLM service is an integrated ICT platform which combines state-of-the-art cognitive exercises with physical activity in the framework of an advanced AAL environment (Konstantinidis et al., 2010). LLM proved the capacities for improving the mental and physical condition of the elderly that adhered to it. The results from 322 individuals participated in the project shown an improvement in the working and episodic memory, as well as in the executive functions (Bamidis et al., 2015). Moreover, 70 elderlies with MCI were subjected to electroencephalographic (EEG) recording in a resting state to investigate whether or not the combination of cognitive and physical activity, induces changes in brain cortical activity. The results revealed a significant reduction in the delta, theta and beta brain rhythms, and a decrease in the activity of certain brain regions (PCU/PCC) associated with functional plasticity. They also discovered that the greater the decrease in the delta and the theta rate, the higher the neuropsychological assessment through Mini Mental State Examination (MMSE; Styliadis et al., 2015). Moreover, graph analysis in brain networks demonstrated the ability of the brain to be “educated” in order to reorganize the functional connectivity to prevent the effects of neurodegenerative phenomena and normal aging (Klados et al., 2016). An additional study was conducted to investigate whether the combination of physical and mental exercise may lead to the enhancement of the brain functional reorganization in the beta band which is considered to be particularly important for mental functioning. Also, an increase in the functional connection of two brain hemispheres of occipital, temporal, parietal and pre-frontal lobe has been occurred (Frantzidis et al., 2014a).

The aforementioned approach is based on a combination of computerized cognitive and physical training, which is performed intensively (more than four times) for a period of approximately 2 months. Both cognitive and physical training are widely used to induce neuroplasticity on the mature human brain and to delay or even prevent neurodegeneration (Heyn et al., 2004; Voss et al., 2010; Frantzidis et al., 2014b; Bamidis et al., 2015). However, there is an active debate on the optimal intervention design and its effect size and sustainability of the positive effects observed. It seems that improvement is greater when the intervention is performed in day care centers under supervision and not at home installations (Lampit et al., 2014). Similarly, multi-domain physical intervention demonstrated improvement or at least preservation of an adequate cognitive functioning in senior citizens (Ngandu et al., 2015). Combination of cognitive and physical training was proven to induce a more pronounced neuroplasticity effect (Frantzidis et al., 2014b; Bamidis et al., 2015). Although, the aforementioned studies demonstrated the applicability of cognitive and physical interventions as well as the robustness of their combination, the research community recently investigates new training approaches which would be more ecologically valid and applicable on non-clinical settings in order to further enhance the adherence rate and to improve drop-outs. Given also the fact that beneficial effects of the training are evident for a given time period after the intervention’s cessation, novel approaches should be adopted as a life-style habit and be performed on a regular basis.

An ideal, non-pharmacological intervention that fulfils the aforementioned criteria is dance. A previous study showed 7% lower risk for developing dementia for elderly people who involved in dancing, playing a musical instrument, reading and playing board games once a week (Verghese et al., 2003). Previous research studies have investigated the potential benefits of dancing interventions to senior citizens. Dance is a less-threatening exercise for many elderlies, since they had many positive and enjoyable experiences in their youth. Also, dancing is an important factor for health and well-being aging. Dance offers the opportunity to maintain a connection with everyday life as it encourages fun and enjoyment, social interaction, team spirit, health, physical activity and mobility. Dance, is considered to be a form of physical exercise that may be adopted as part of many interventions for the elderly instead of other (Wikström, 2004; Lima and Vieira, 2007). Dance improves energy, increases mood and reduces stress or anxiety in ways similar to aerobic exercise (Kim and Kim, 2007). Also, studies have reported that the traditional dances as a physical activity prevent cognitive decline and improve the coordination and control of body movements (Balkus, 1989).

Dance training has been associated with positive behavioral effects in various populations. However, only few studies have evaluated the functional brain correlations in dance interventions as they have not investigated the structural correlations of brain changes due to the dance exercise. It is necessary to perform additional research on brain related networks and correlative behaviors of dance interventions, so as to better understand and validate the dance-related neuroplasticity mechanisms. Thus, there is a huge need to further study the neuroscience of dance in order to provide an increasing multidisciplinary area that may offer information on the interactions between the brain and arts (Karpati et al., 2015).

Thus, traditional dancing may induce many feelings as it is achieved in groups of 15–20 participants who simultaneously perform dancing movements which require balance, flexibility, rhythm, orientation and cognitive functions. More even it does not require any special equipment and can be applied easily by Day Centers with low cost.

Despite the promising results, the majority of studies that are employed dancing training have not been evaluated under medical and neuroscientific criteria. Therefore, we studied an experimental group who performed traditional dancing for 6 months. This group was compared with a control group which performed a cognitive stimulation program. Both groups were assessed in terms of a detailed neurophysiological assessments and neurophysiological (EEG) examination which aimed to assess the brain functioning of the participants before and after the training. This examination evaluates the brain functions in terms of each functional organization through a system level approach employed analysis of brain networks based on graph theory.

This piece of work aims to investigate whether traditional dancing could improve cognitive and brain functioning of elderly compared with a cognitive stimulation program (Active Control Group). We also attempt to answer the following research questions:

- Is traditional dancing intervention an effective approach for promoting active and healthy aging?
- Which are the brain alterations induced by dancing?
- Which is the type of such alterations?

According to previous studies we hypothesize that traditional dancing may be more effective than active stimulation. It would also induce functional re-organization of the brain networks that would shift brain function to a more optimal mood. The latter is hypothesized to be achieved by more efficiency and faster communication among distant brain regions.

## Materials and Methods

### Participants

The study initially employed 54, non-demented, senior citizens, who were randomly assigned either to an active control (mean age 66 ± 5.51 years) or to a traditional dance training group (mean age 68.73 ± 4.73 years). However, five participants from the training-group refused to repeat the neurophysiological intervention at the post level and they excluded from the study. There were also five participants who did not complete the active control program and also excluded. So, the study resulted in 44 senior citizens. Both groups consisted of equal number of participants. Neuropsychological, physical status evaluation and neurophysiological by an electroencephalogram (EEG) were performed. The evaluation took place 1–14 days before/after the training initiation/finalization respectively. Prior to their enrolment in the study activities all participants were informed about the study hypotheses and aims as well as the underlying activities. Inclusion criteria were the following: (1) age to be greater than 60 years; (2) participants should be sedentary or mild sedentary with an upper threshold of 150 min of moderate to vigorous exercise per week; (3) lack of participation in any other physical training intervention within the last year; and (4) performance of medical examination assessing their safe participation in the study. People with heart failure, hypertension and respiratory insufficiency and patients suffering from dementia were excluded from the program as they who participated in other Greek Traditional Dances programs. Also, participants who did not complete at least 80% of the total of hours of program were excluded from further analysis. They had the opportunity to discuss any issues raised and then they signed a written informed consent form. The study was based on specific guidelines and regulations, which were approved by the ethics committee of the Aristotle University of Thessaloniki (AUTH) according to international principles. Group demographics and information about the participants’ age, education, body mass index (BMI) and cognitive status, is depicted in Table 1. The groups did not significantly differ in baseline cognitive level, mean age, education and gender (all *p*s > 0.05).

**Table 1:** Number of participants per intervention group, age, education, Body Mass Index (BMI) and cognitive status as measured by the Mini Mental State Examination (MMSE), Trail B and Geriatric Depression Scale (GDS).

	Active group *n* = 22		Dance group *n* = 22		*p*-value
	Mean	SD	Mean	SD	
Age (years)	66	5.51	68.73	4.73	0.091
Education (years)	8.64	3.79	9.55	4.63	0.481
% Female	77.3		95.5		
BMI	30.14	2.92	29.10	4.90	0.408
MMSE	26.73	2.88	27.05	2.75	0.710
Trail B	146.30	51.99	194.11	82.33	0.036
GDS	2.76	3.0	2.00	2.6	0.427

### Intervention

The active group participated in cognitive intervention with the use of Video GRade software that was developed by the Lab of Medical Physics at the AUTH. Video GRade is an inspired environment for cognitive training^1^, which consists of a set of 50-min educational video from YouTube that includes content with history, art, culture, dance and music. At the end of each video, participants were asked to answer eight multiple choice questions. Participants performed 24 sessions during a period of 8–10 weeks.

The intervention group was conducted through traditional dances from all over Greece that were divided into three categories according to the number of steps and complexity, the intensity of the pace (slow-fast), as well as the upper limbs position and movement. A 24-week period program was performed for 60 min two times per week, taking place at Day Care Centers of Municipality of Thessaloniki located in Greece. The intervention duration was much longer than the active (Video Grade) control group, due to the annual organizational structure of the local Day Care Centers. So, the maximum number of available sessions was 48. However, the long duration of the intervention overlapped with Christmas and Easter holidays. During that period, many senior citizens preferred to stay at home and did not attend 1–4 (maximum two successive weeks) sessions per occasion. Considering the abovementioned pragmatic limitations and aiming to have a similar number of sessions between the control and the training group we kept the mean number of 28 sessions within the 24-week period.

### Neuropsychological and Physical Assessments

Cognitive functioning was estimated through the MMSE (Folstein et al., 1975), Trail Making B Test (Butler et al., 1991) and the Geriatric Depression Scale (GDS short version; Yesavage et al., 1982–1983).

Their physical status and functional ability was evaluated through the following tests: (i) Fullerton Senior Fitness Test which contains six domains: chair Stand (assessed lower body strength), 8 Foot Up and Go (assessed complex coordination, agility and dynamic balance), Back Scratch (assessed flexibility of upper body), Chair Sit and Reach (assessed flexibility of the lower back and hamstring muscles), Arm Curl (assessed upper body strength) and 2 Min Step (assessed aerobic capacity; Jones and Rikli, 2002); (ii) Berg Balance Scale which assessed balance and risk of falls (Berg et al., 2009); (iii) Tinetti Test which assessed walking and risk of falls (Tinetti, 1986); and (iv) Stork Balance Stand Test which assessed balance when standing on one leg (Johnson and Nelson, 1969). Body composition was also calculated.

### EEG Analysis

#### Data Acquisition

**Figure 1:**
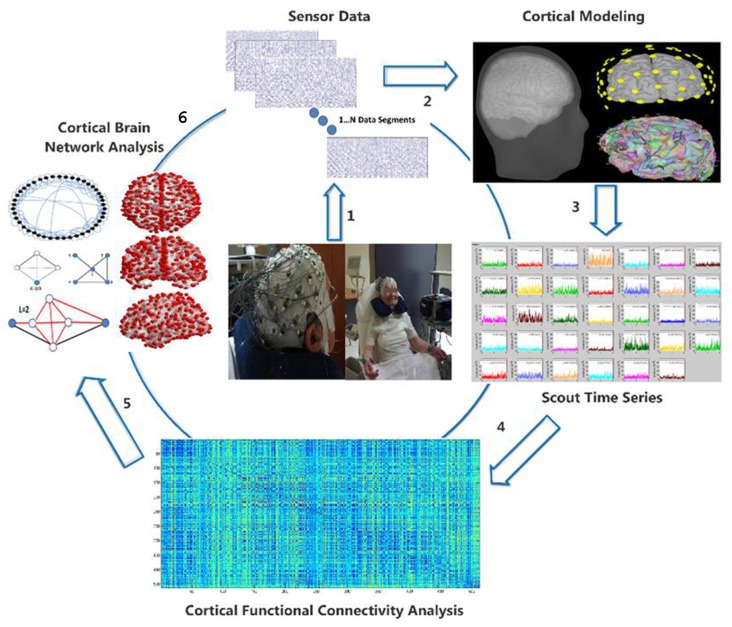
Visualization of the electroencephalographic (EEG) methodology from transforming the raw EEG data (1) to the cortical activity (2), estimation of regions of interest (ROIs) cortical activity (3), quantification of functional connectivity matrix (4) and finally cortical brain network analysis (5) through graph theory (6).

The EEG data acquisition was performed through a Nihon Kohden JE-207A device and 57 active electrodes attached on a cap (EASYCAP) fitted directly to the participant’s head. Two more electrodes were placed on positions behind the participant’s ears and a third active electrode, placed on a left anterior position, served as ground electrode. The electrode impedances were kept lower than 5 KΩs. The sampling frequency was set at 500 Hz. The participants sat on a comfortable, armed chair, which was located in a quiet room with minimal, ambient light. They were instructed to close their eyes for 5 min and avoid any movement, especially of their upper part. The subsequent processing and analysis steps are visualized in Figure 1 and are the following: (i) electroencephalographic (EEG) data acquisition; (ii) artifact removal and sensor data epoching; (iii) generic anatomy head and cortex modeling; (iv) estimation of the cortical activity for anatomically relevant regions of interest (ROIs); (v) quantification of the co-operative degree (functional connectivity analysis) among ROIs cortical activity through the notion of Relative Wavelet Entropy (RWE); and (vi) formation of functional brain networks regarding the ROIs as network nodes and their connectivity values as edges. The analysis is performed through contemporary mathematical tools derived from graph theory and involves both global and local network properties.

#### Pre-processing

The 57 active electrodes recording brain signals were re-referenced through the common average model. Then, digital filtering through 3rd order Butterworth filters was applied as follows: (a) high-pass filter with cut-off frequency at 1 Hz; (b) band-stop (notch) filter with cut-off frequency at the 47–53 range; (c) low-pass filter with cut-off frequency at 100 Hz; (d) band-stop (notch) filter with cut-off frequency at the 97–103 Hz range; and (e) band-stop (notch) filter with cut-off frequency at the 147–153 Hz range. Independent Component Analysis was then applied to identify and reject potentially contaminated source components due to eye blinks, muscle artifacts, bad electrode placement, high frequency noise, ECG modulation, linear trends. Finally, the data were epoched. The epoch duration was 1,024 sample points (2.048 s). Epochs still containing artifacts were visually rejected. The pre-processing was performed through built-in code based on the Matlab Signal Processing Toolbox and EEGLAB software (Delorme and Makeig, 2004).

#### Cortical Activity Estimation

The Brainstorm software package was used for the computation of the inverse problem, which results in the reconstruction of the cortical activity (Tadel et al., 2011). Default anatomy modeling was employed in terms of magnetic resonance imaging (MRI) volume (Colin 27 stereotaxic T1-weighted MRI volume). The head modeling was computed through the Open MEEG Boundary Elements Method (BEM head model). The solution space was constrained to the cerebral cortex which was modeled as a 3-dimensional grid of 20,000 fixed dipoles oriented normally to the cortical surface. Then, the inverse solution was estimated by means of the sLORETA methodology (Pascual-Marqui, 2002). The study analysis, employed the entire cortex analysis through the definition of 512 cortical regions of interest (ROIs), which were defined according to the deterministic process implemented by the Brainstorm software. Since, the source analysis requires a symmetric electrode map of 64 electrodes, seven additional electrode activations were inserted through first-neighborhood activity interpolation.

#### Cortical Synchronization Analysis

The synchronization analysis aims to quantify the co-operative degree among the ROIs by comparing their cortical time series activity. Therefore, it was performed for each participant on the average scout cortical activity. The Orthogonal Discrete Wavelet Transform (ODWT) was applied and the analysis was based on the relative wavelet entropy (RWE) metric. Bi-orthogonal wavelets of 5th order were selected as the family wavelet class (Frantzidis et al., 2010, 2014b; Chriskos et al., 2018). Optimal time-frequency analysis is obtained since the mother wavelet is subjected to scaling and translation. The ODWT analysis framework is based on an iterative decomposition scheme, which employs recursive, low-pass filters for estimating the activity of the five frequency bands (delta, theta, alpha, beta and gamma) through the estimation of the wavelet coefficients. The decomposition scheme involves *j* = 1…5 layers. The amplitude of the wavelet coefficients quantifies the wavelet’s correlation with the actual rhythmic activity, while the coefficient’s sign is an index of either positive or negative correlation. The ODWT framework involves a periodization mode. So, the final decomposition layer (*j* = 5) contains one wavelet coefficient and corresponds to the lowest rhythms (delta and theta). Each other step contains the double number of the previous one. So, there are two coefficients for the alpha, four for beta and eight for the gamma rhythm. In this way, non-redundancy and optimal resolution is facilitated through a parameter-free methodology. The energy of each frequency band is estimated by squaring the amplitude of the corresponding wavelet coefficients. Then, the overall energy is estimated as following:

(1) $$E_{tot}=\sum_{j<0}\sum_{k}{\left|C_{j}(k)\right|}^{2}$$

In this way, the relative energy distribution of each frequency band is obtained and the synchronization degree among two ROIs was quantified as the similarity of their energy distribution (*p*_j_ and *q*_j_, *j* = 1…5) given by the following formula of the RWE:

(2) $$RWE=\sum_{j=1}^{5}p_{j}\times 1\text{n}\left(\frac{p_{j}}{q_{j}}\right)$$

The RWE metric may be then used as a dissimilarity matrix (the larger the value the greater the desynchronization), which may be used as an adjacency matrix for network analysis through graph theory.

#### Cortical Brain Network Analysis

##### Functional Connectome Estimation

For each participant and for each time condition (pre/post-intervention) all the available data epochs were used. For each epoch the resulting RWE matrix was used to create a functional brain network, which is defined as a graph G = (V, E). The graph contains a set V of nodes (here *V* = 512 cortical ROIs) and a number E of edges quantifying the co-operative degree as estimated through the RWE analysis. The RWE matrix is used as it is, but the auto-synchronization information of the main diagonal is discarded from further analysis. Therefore, the resulting adjacency matrix forms a weighted and directed network. Subsequent analysis was performed on binary networks. The thresholding was dynamically configured for each network instance in order to result in fixed density ranges of 10,000, 12,500 and 15,000 edges. The density range was similar as in previous studies (Frantzidis et al., 2014a).

##### Global Network Characteristics

Global network analysis was applied by computation of both the cluster coefficient for each node and the characteristic path length for each pair of ROIs. Then, their mean values were used for the estimation of the small-world property. While, the interested reader may find detailed information on these fundamental network properties in classical textbooks and studies, in brief the cluster coefficient is an index of local information processing, while the characteristic path length quantifies the integration of information and the network’s flow. More specifically, the cluster coefficient (C) for a given node is the number of the existing connections of the node’s direct neighborhood to the total number of the possible ones. The set of the node’s direct neighborhood contains the ROIs with which the node under consideration is connected. So, C is actually a possibility of the local information capacity for each node. The characteristic path length (L) is applied on node pairs and quantifies the shortest distance among those nodes. The mean values of these two characteristics are computed for both the actual brain network and for a number (*N* = 100) of random networks with the same characteristics (nodes and density). Then, the small world property (sigma) is computed as follows:

(3) $$sigma=\frac{\frac{L}{L_{\text{rand}}}}{\frac{C}{C_{\text{rand}}}}$$

In the case that the mean cluster coefficient of the actual brain network is much larger than the mean value of the random ones and simultaneously the characteristic path length of the actual and the random networks are quite similar, we have sigma >1. The greater the sigma the more prominent the small-world property is. According to these, small-world is an index of non-random clustering and near-random path length. So, it is a property of the optimal network configuration since it implies strong local information processing which may be transferred from every individual node to every other, regardless of their physical distance, through a small set of intermediaries (Watts and Strogatz, 1998).

##### Brain Hub Detection

Functional hubs of the cortical connectome were identified based on the betweenness centrality (BC) metric (Bi). It is an index of the information amount transferred through a given node, since it is computed as the number of the network’s shortest paths that pass through the specific node (Rubinov and Sporns, 2010). Hub detection was performed based on a criterion of Bi ≥1.5, which was firstly introduced by Seo et al. (2013) and previously applied by Frantzidis et al. (2014b).

##### Functional Cartography and Node Roles

Aiming to ensure that putative effects were not biased by the particular hub definition, we also followed an alternative approach of applying a previously proposed functional cartography technique (Guimerà and Nunes Amaral, 2005). This framework involves the computation of the community structure (modularity analysis) and then the estimation of the within-module z-score and the participation coefficient (PC). Based on these two metrics a specific role is assigned to each network node. The network’s community structure was performed based on the Brain Connectivity Toolbox (BCT; Rubinov and Sporns, 2010). Then, the within-module z-score was used to estimate the connection strength of a given node with the other nodes of its own module. The greater the z-score is, the larger the connection strength. The PC (P) is a probability value, which quantifies the connection distribution of a given node across all the feasible modules of the specific network. The closer the coefficient’s value to 1, the more uniform the distribution across modules is. The role assignment to each node is defined based on the node’s position to the *z*-P parameter space. Heuristically, in case a node has within-module z-score greater or equal to 1.5 then it is regarded as a hub and otherwise as non-hub node. Both node categories are then further characterized according to their PC value. More specifically non-hubs are further characterized as ultra-peripheral (*p* ≤ 0.05), peripheral (0.05 < *p* ≤ 0.45), connector (0.45 < *p* ≤ 0.70) and kinless (*p* > 0.70). Hub nodes are assigned as provincial (*p* ≤ 0.25), connector (0.25 < *p* ≤ 0.50) and kinless (*p* > 0.50).

## Results

### Analysis of Global Network Characteristics

The statistical analysis was conducted *via* a 2 × 2 mixed model ANOVA. The groups of intervention (Dance and Active) served as between-subjects factor whereas the time (pre-post) as within-subject factor and the three inter-related dependent variables (small-world value, cluster coefficient and characteristic path value).

**Figure 2:**
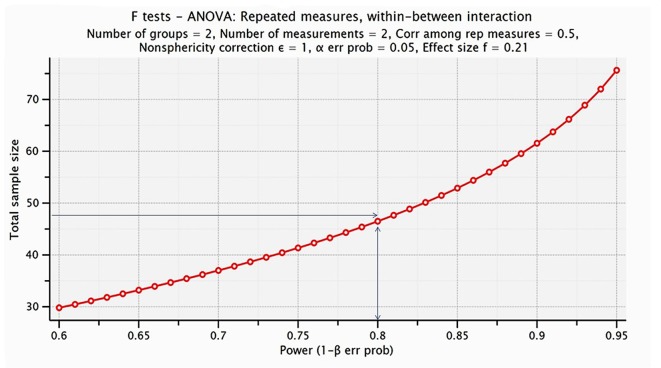
Visualization of the analysis performed for estimating the proper sample size for the proposed statistical analysis. The power analysis indicated that the proper sample size was 48 (24 participants per group), whereas we have included 44 senior citizens (22 participant per group).

Aiming to investigate whether the sample size was adequate for the statistical analysis we performed power analysis. It was conducted in G-Power 3.1 to determine a sufficient sample size using an alpha of 0.05, a power of 0.80, and a medium effect size (*f* = 0.21; Faul et al., 2013). Based on the aforementioned assumptions, a total sample size of 48 people would be sufficient to detect significant interaction effects. Specifically, the appropriate number for each group of intervention (Dance and Active) is 24, in order for group differences to reach statistical significance at the 0.05 level. Our sample size is estimated 44, 22 per group which is adequate for our experimental design and the analysis performed. The results obtained from the statistical analysis are visualized in Figure 2.

Results revealed an interaction between time and intervention for the small world property for 10,000 edges, *F*_(1,42)_ = 20.56; *p* < 0.05, as well as the small world property for 12,500 edges *F*_(1,42)_ = 19.53; *p* < 0.05. Furthermore, an interaction between time and intervention was also revealed for the characteristic path for 15,000, *F*_(1,42)_ = 5.16; *p* < 0.05 as you can see in Table 2.

**Table 2:** 2 × 2 mixed model ANOVA statistical analysis.

Variables	Edges	*F*_(1,42)_	Time/Interv.	Between intervention
Small world value	10,000	20,565	**0.00**	**0.033**
	12,500	19,530	**0.00**	0.19
Characteristic path	15,000	5,163	**0.028**	**0.005**

*The groups of intervention (Dance and Active) served as between-subjects factor whereas the time (pre-post) as within-subject factor and the three inter-related dependent variables were the small-world value, cluster coefficient and characteristic path length. The p-values that reached statistical significance (*p* < 0.05) are displayed in bold*.

### Correlation With Cognitive and Physical Status

Pearson’s correlations were computed, in order to test if there was a linear relationship between cognitive status as measured with the MMSE assessment and physical status as measured with the Fullerton Senior Fitness Test, as well as the Small-World property value. The analysis revealed a significant positive correlation between the Back-Scratch Test score and the Small World property at 12,500 edges, *r* = 0.301, *n* = 43, *p* = 0.05 and between the Chair Sit and Reach Test score and the Small World property at 15,000 edges, *r* = 0.385, *n* = 43, *p* = 0.01. Furthermore, a significant negative correlation was also revealed between the Back-Scratch Test score and the PC of the fronto-parietal network (FPN) score, *r* = −0.32, *n* = 43, *p* = 0.039.

### Analysis of Physical Status Assessments

A paired-samples *t*-test was conducted to investigate the effectiveness of Greek traditional dancing on dependent measures of functional connectivity. Table 3 presents the results (post–pre-intervention differences and *p*-value) concerning the physical assessment tests. More specifically, the results indicate that the intervention group evoked statistically significant improvement in the Chair Stand, Chair Sit and Reach and 8-Foot-Up-and-Go. Active Control group showed statistically significant improvement in Chair Stand test.

**Table 3:** Statistically significant changes of each group regarding the Fullerton examination.

TEST	Subdomain	Active group (*N* = 22)		Dance group (*N* = 22)	
		*t*_(20)_	*p* value	*t*_(21)_	*p*-value
Fullerton	Chair stand	−3.700	**0.001**	−3.926	**0.001**
	Arm Curl	−1.407	0.175	−1.588	0.127
	2-min walk in place	0.541	0.595	−0.606	0.551
	Chair sit and reach	−1.494	0.151	−3.023	**0.006**
	Back scratch	−0.583	0.567	−1.648	0.114
	8-Foot-Up-and-Go	1.303	0.207	3.925	**0.001**

*Bold values denote statistical significance*.

### Local Network Analysis

From the 512 ROIs we further analyzed the 22 being part of the default mode network (DMN), FPN, and executive network (EN) according to Voss et al. (2010). For these three networks as well as for their combination we estimated the following local graph properties: PC, BC and within module z-score (ZM). We selected these metrics in order to define node hierarchy according to Guimerà and Nunes Amaral (2005) and Seo et al. (2013). The statistical analysis was conducted *via* a 2 × 2 mixed model ANOVA. The groups of intervention (Dance and Active) served as between-subjects factor whereas the time (pre-post) as within-subject factor and the three inter-related dependent variables (BC, PC and within Module z-score value). Table 4 presents the results that revealed an interaction between time and intervention for the EN for the BC *F*_(1,42)_ = 23.246 *p* < 0.05 and Within module z-score *F*_(1,42)_ = 37.262 *p* < 0.05. Furthermore, an interaction between time and intervention was also revealed for FPN for BC *F*_(1,42)_ = 9.291 *p* < 0.05, PC *F*_(1,42)_ = 25.695 *p* < 0.05 and Within module z-score *F*_(1,42)_ = 22.284 *p* < 0.05. Finally, PC was also statistically significant feature for DMN *F*_(1,42)_ = 7.740 *p* < 0.05 and contribution of all *F*_(1,42)_ = 11.069; *p* < 0.05.

**Table 4:** Statistically significant changes regarding local network (betweenness centrality, participation coefficient, within-module z-score) at pre-post level for both groups.

Network	Features	*F*_(1,42)_	Time/Interv.	Between intervention
Executive	Betweenness centrality (BC)	23.246	**0.000**	0.164
	Participation coefficient (PC)	0.219	0.642	0.079
	Within module z-score (ZM)	37.262	**0.000**	0.695
Fronto-parietal	Betweenness centrality (BC)	9.291	**0.004**	0.364
	Participation coefficient (PC)	25.695	**0.000**	0.490
	Within module z-score (ZM)	22.284	**0.000**	0.494
Default mode network (DMN)	Betweenness centrality (BC)	0.860	0.359	0.068
	Participation coefficient (PC)	7.740	**0.008**	**0.002**
	Within module z-score (ZM)	1.450	0.235	0.417
Contribution of all	Betweenness centrality (BC)	1.926	0.172	**0.001**
	Participation coefficient (PC)	11.069	**0.002**	**0.020**
	Within module z-score (ZM)	3.286	0.077	0.180

*The p-values that reached statistical significance (*p* < 0.05) are displayed in bold*.

#### Hub Identification

**Figure 3:**
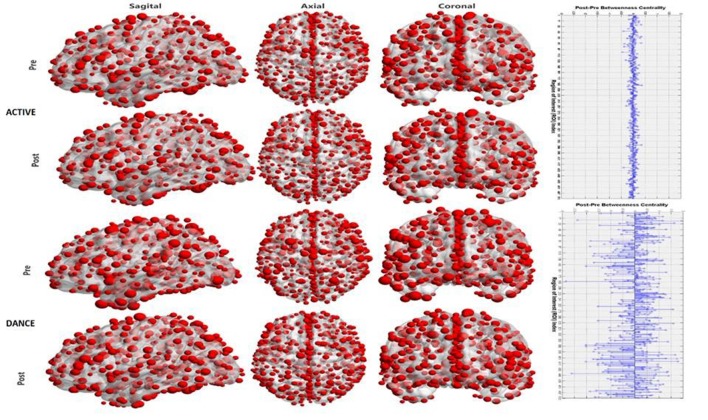
Visualization of the hub significance of each node in terms of betweenness centrality (BC). The first column (from the left) displays a sagittal view of the cortex, the middle one an axial view and the right one a coronal view. Rows 1–2 are for the active control group for pre and post conditions respectively. Rows 3–4 are for the Dance group. The right plot denotes the pre-post differences in the hub strength for the active (upper plot) and for the dance (lower plot).

As described within the “Methodology” section (Brain Hub Detection), hubs were identified according to their relative BC value (B_i_). The B_i_ value for both groups and both conditions as well as their post-pre/intervention difference are visualized for each cortical node in the Figure 3.

#### Functional Cartography Analysis

**Figure 4:**
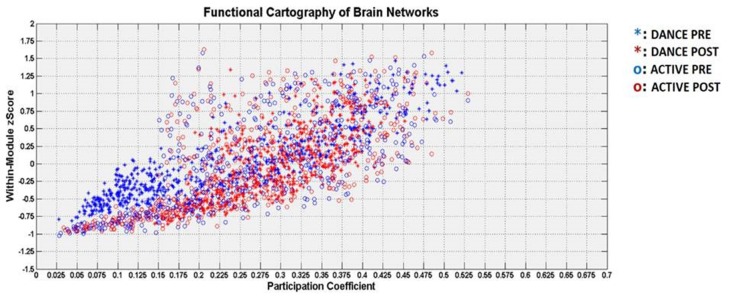
Visualization of the functional cartography in terms of participation coefficient (PC; horizontal axis) and within-module z-score (vertical axis). The DANCE group is denoted with asterisk and the ACTIVE group with circle. The pre-intervention is denoted with blue color and the post condition with red color.

The Figure 4 describes the two-dimensional distribution in terms or PC and within-module z-score for each cortical node, for both groups and conditions.

**Figure 5:**
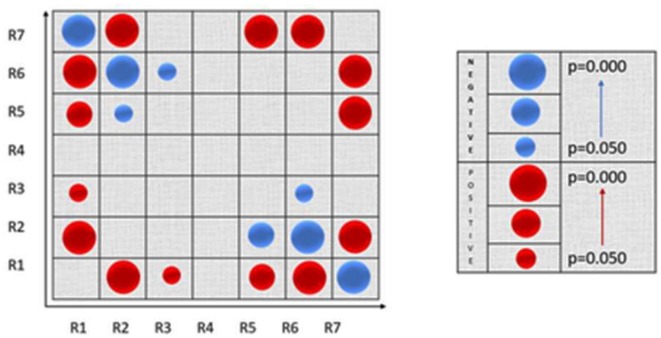
Visualization of the Pearson correlation among nodes’ roles. Positive correlations are denoted with blue color and negative correlations with red color. The circle’s size is proportional to the strength of the statistical significance.

Pearson analysis revealed statistically significant correlations among the node roles (both positive and negative) as visualized in the following Figure 5.

## Discussion

This study investigated dancing induced neuroplasticity effects of the mature human brain. We employed an objective evaluation framework based on contemporary mathematical tools such as cortical functional connectivity and graph theory. Network neuroscience has been applied until now to quantify physiological aging mechanisms (Micheloyannis et al., 2009) and neurodegeneration phenomena (Sanz-Arigita et al., 2010; Tijms et al., 2013; Frantzidis et al., 2014a). However, there are only a few preliminary attempts on how cognitive and physical training improved brain network functioning (Burdette et al., 2010; Voss et al., 2010; Klados et al., 2016). The pioneering work of Voss et al. (2010) employed functional MRI (fMRI) and showed that aerobic training increases the functional connectivity of the Default Mode and the Fronto-Executive resting state network. The authors found that this type of intervention may increase restoration mechanisms in fronto-temporal, brain networks affected by physiological aging. However, they did not investigate how aerobics improve the functional organization of these networks as an entire system. A similar type of training was also employed by Burdette et al. (2010) and validated that physical exercise increases hippocampal connectivity in comparison to a control group. The authors employed graph theory tools to evaluate these changes and showed that physical training enhanced the hippocampus degree and its connectivity with the anterior cingulate cortex. These two regions were part of the same sub-network (module) for the training group. A more recent work by Klados et al. (2016) examined whether a combination of computerized cognitive and physical training would induce changes in a cortical functional network. They evaluated these changes in terms of alterations in network’s density and node’s strength. They focused on beta band and on cortical estimations of 305 regions defined by sLORETA software.

Despite the impact of these three studies, they introduced only a partial estimation of the network properties associated with neuroplasticity. Due to methodological limitations attributed either to the low temporal resolution of fMRI (Burdette et al., 2010; Voss et al., 2010) or to the cortical estimation employed by Klados et al. (2016), the previous studies failed to track a global perspective of how training changed the cortical functional organization of the mature human brain. Moreover, the local analysis they performed was minimal since it focused only on modularity analysis of central brain regions (Burdette et al., 2010) or node strength in beta band (Klados et al., 2016). Although these analyses were significant since they shed light at the prominent role of hippocampus and anterior cingulate cortex (Burdette et al., 2010) and the hub strength of cortical regions (Klados et al., 2016) they did not provide answers whether physical intervention increases local information processing or information flow resulting thus in better functional organization as estimated by small-world property (Watts and Strogatz, 1998; Frantzidis et al., 2014a). Moreover, the hub definition through node strength as defined in Klados et al. (2016) is mainly a connectivity-driven metric that may also incorporate compensatory mechanisms and did not regard the node’s contribution to the global information flow. Therefore, we employed the BC metric as defined in Seo et al. (2013) and Frantzidis et al. (2014a). Finally, we proposed a more detailed network analysis through the notion of functional cartography as defined in the article of Guimerà and Nunes Amaral (2005). We used that type of analysis to assign to each cortical node a specific role, but we also investigated correlations (either positive or negative) among these nodes and how intervention changes the node distribution to these specific roles. The network analysis was applied to a detailed cortical parcellation (512 nodes) but also in known resting state networks (DMN, FPN and EN) as defined in Voss et al. (2010). So, we believe that the main novelty of our study is the proposal of a holistic intervention evaluation framework based on network neuroscience and covering most of the aspects of graph theory.

Our results regarding global network characteristics demonstrated significant time by intervention interactions for both small-world metric (in density ranges of 10,000 and 12,500 edges) and for characteristic path length regarding 15,000 edges. This was because the participants in dance training showed a significant increase of small-world property at two density ranges mainly attributed to a decrease of characteristic path length which was significant only for 15,000 edges. Although there are not previous global network results regarding intervention evaluation, there is a plethora of studies reporting disrupted functional organization of brain networks due to neurodegeneration. More specifically, the pioneering work of Stam et al. (2008) showed that both cluster coefficient and characteristic path length obtained through Phase Lag Index (PLI)-weighted connected networks were decreased for AD patients. Similarly, sensor-based EEG analysis showed decreased small-world property in the AD group within the beta band (de Haan et al., 2009). The first fMRI-based study performed by Supekar et al. (2008) showed loss of small-world property in AD patients due to decrease in local information processing (cluster coefficient). Another study through fMRI found that AD patients showed increased cluster coefficient and characteristic path length in comparison with a healthy control group (Zhao et al., 2012). Finally, a recent study employing healthy elderly and patients suffering either from amnestic MCI (aMCI) or from mild dementia (MD) reported functional disorganization of small-world brain networks for both pathological groups (Frantzidis et al., 2014a). This decline was mainly attributed to the loss of local information processing capacity (mean cluster coefficient). The aforementioned studies indicate that functional brain networks are vulnerable to neurodegeneration phenomena, which affect brain networks through a multi-level way. The proposed dancing intervention seems to improve functional network performance as estimated through small-world property by reducing the average length of characteristic paths. This implies minimization of functional disconnection patterns and better transmission of the information across network nodes. This finding is of particular importance since Alzheimer’s dementia is regarded as a disconnection syndrome resulting thus in longer characteristic path lengths (Stam et al., 2007; Frantzidis et al., 2014a). The specific hypothesis is supported by previous studies which linked longer path lengths and preserved cluster coefficient with a loss of complexity and a less optimal organization (Stam et al., 2007). Among the first findings of the network neuroscience were that increases of characteristic path length induce functional degradation of brain network and reduced interaction among brain nodes (Sporns and Zwi, 2004) which was also correlated with cognitive decline (Stam et al., 2007). So, the present study results regarding global network analysis (increase of small world property due to preservation of cluster coefficient and reduction of characteristic path length) may be attributed to neuroplasticity findings due to the dance intervention which seems robust for promoting active and healthy aging.

We also investigated whether the improvement of physical activity may be correlated with neuroplasticity effects. The study demonstrated positive correlations among the small-world property and the *Back Scratch* (*r* = 0.301, *p* = 0.05) and *Chair* *Sit and Reach* (*r* = 0.385, *p* = 0.01) domains of the Fullerton Senior Fitness test. These two tests quantified the flexibility of the upper and the lower body respectively. Flexibility is regarded as an index of functional status of the elderly and could be benefited from physical training (Heyn et al., 2004). Dance training induces greater improvement on trunk flexibility and hip-joint mobility (Sofianidis et al., 2009). According to that study, traditional Greek dancing seems to be an ideal candidate for improving flexibility since it involves activities of single-limb standing, while the dancer’s body should be flexed/extended, and many steps require forward/backward leaning (Sofianidis et al., 2009). However, previous studies have not investigated how such an intervention affects the functional organization of brain networks. Positive correlations imply that flexibility improvement may be induced by a more efficient function of the mature human brain as quantified by the small-world property. This finding is novel since it identified for first time a positive relationship among flexibility increase and global network performance.

Although, positive correlation of physical activity with global network characteristics is a novel finding which has not been earlier demonstrated to the best of our knowledge, it does not provide information about the mechanism that traditional Greek dance induces neuroplasticity. Aiming to further investigate this issue we performed local network analysis by investigating functional cartography (Guimerà and Nunes Amaral, 2005) and hub identification (Seo et al., 2013; Frantzidis et al., 2014a). This analysis was performed in order to define the functional node hierarchy both on the entire set of nodes and on specific resting-state (Default Mode, Fronto-Parietal and Executive) networks according to (Voss et al., 2010). We computed the BC as a generic hub index, PC as an index of how well a node is connected with nodes belonging to other modules and within-module z-score which quantifies the connection strength of a given node with the other nodes of its own module. The previous finding of positively correlating flexibility improvement with better functional organization (small-world increase) was further validated by the negative correlation observed among the *Back Scratch* and the PC for the nodes of the FPN (*r* = −0.32, *p* = 0.039). FPN seems to be greatly affected by the proposed intervention since there were also statistically significant changes in BC, PC and within module z-score for that network. FPN functioning was linked with goal-directed attention (Uddin, 2015), motor planning and execution (Hsu et al., 2017). It was also shown that fitness gains were associated with greater activation of the FPN and better executive functioning (Colcombe et al., 2004). Combining the previous neuroimaging findings with our results regarding the re-organization of the fronto-parietal and executive resting state network, we may conclude that traditional Greek dance is a potentially robust approach for improving the flexibility of senior citizens. This was achieved by inducing neuroplasticity effects on the modular architecture of resting-state networks known to be affected by increased physical activity (Colcombe et al., 2004; Voss et al., 2010). These networks were also previously demonstrated to govern complex motor planning and executive functioning resulting thus in improved flexibility which is essential during dancing. Neuroplasticity was also evident and for the DMN, as denoted by PC changes, but its effect was more attenuated in comparison to the aforementioned networks.

The aim of the present study was to examine whether a program of traditional Greek dance could induce neuroplasticity effects on the mature human brain. Dancing involves learning of motor skills through physical practice and observation (Kirsch et al., 2018), while it combines cognitive and physical training (Rehfeld et al., 2018). Although it involves a moderate level of physical activity, poses several sensorimotor and cognitive challenges. It also facilitates social interaction enriched with emotional stimulation (Kattenstroth et al., 2013). So, dancing is regarded as a promising intervention for promoting neuroplasticity, healthy and active aging (Merom et al., 2016). The pioneering work of Kattenstroth et al. (2013) demonstrated positive effects on posture, reaction time, cognition, motor performance and quality of life, when compared with a control group. Despite their novelty, the aforementioned studies did not investigated changes in terms of functional or structural neuroimaging, even though dance often involves the activation of premotor, parietal and occipito-temporal cortices (Vogt and Thomaschke, 2007). However, these brain regions are known to be affected by aging mechanisms, resulting thus in poor performance during complex motor sequences like those required for dancing (Kirsch et al., 2018). Our hypothesis was that an intervention based on traditional Greek dance would challenge mental, cognitive and physical abilities of senior citizens. This would be achieved through music stimuli stemming from their youth and requiring execution of complex motor sequences. During the choreography performance, dancers need to co-ordinate their body posture and movement according to the time-varying music characteristics. This would be achieved by improving their balance, flexibility and strength through the functional re-organization of the mature human cortex. Neuroplasticity effects were evident at a global network level denoted by faster information flow among distant brain regions. This may imply the optimized performance of specific neural networks governing elaborate cognitive processing, balance and entire body co-ordination (Voss et al., 2010). Our results validated previous studies that compared a challenging 6-month dance training program with a conventional fitness intervention of equal intensity (Rehfeld et al., 2018). Although both interventions increased physical fitness, dancing was associated with greater brain volume increase and with an increase in plasma BDNF level (Rehfeld et al., 2018). Longer duration of dance intervention was associated with greater activation of the left hippocampal volume and with improved balance performance (Rehfeld et al., 2017). These studies employed advanced neuroimaging techniques for localizing either structural (Rehfeld et al., 2017, 2018) or functional (Kirsch et al., 2018) changes associated with dancing. Our approach aims to provide an evaluation framework, quantifying dynamical interactions among cortical regions based on contemporary mathematical tools derived from graph theory. So, we focused not only on how the dance training changed the macroscopic functional organization of the human brain, but we also investigated local changes on the default-mode, fronto-parietal and executive resting state networks.

Although our results and their interpretations shed light into the neuroplasticity induced by dancing interventions, they should be considered in the context of several limitations. Firstly, the analysis employed an EEG device with 57 active electrodes. Similar EEG recording settings have been used successfully in previous studies (Styliadis et al., 2015; Klados et al., 2016), nonetheless one should take into account in the interpretation of the results the low spatial resolution of such a setting. High density EEG systems with 128 channels may identify with greater accuracy the cortical activations obtained as outcome measures from similar intervention procedures. Moreover, our brain network analysis was based on a generic head anatomy modeling which could not deal with inter-subject variability and therefore may be vulnerable to errors due to atrophy, life style factors, tissue conductivity or injuries. Given that the local network analysis was based on ROIs defined by a previous neuroimaging study (Voss et al., 2010), estimation of cortical activity for these ROIs may be prune to methodological limitations and results should be interpreted with caution. However, our results identifying a major re-organization of fronto-parietal and ENs are in line with a plethora of previous neuroimaging studies (Colcombe et al., 2004; Voss et al., 2010; Hsu et al., 2017; Kirsch et al., 2018). Future studies may also avoid using thresholding of the connectivity values and employ weighted networks. An important aspect of future research will be the validation of our results with resting state data derived from other recording modalities such as magnetoencephalography (MEG) or fMRI. Another possible limitation is the relatively small sample size of participants we employed. Although, power analysis indicated that the proper number of participants is close to the actual one (48 vs. 44), larger cohorts is expected to provide more definitive results regarding the neuroplastic changes due to the dance intervention. We should also mention that the present study enrolled only Greek senior citizens and lacks diversity concerning ethnic background. Our future aim is to investigate the generalizability of the intervention to other sample cohorts across the world and to compare it with other traditional dance types. Finally, future studies should investigate whether the beneficial effects of dancing are dependent from demographic factors (age, gender), education, cognitive reserve, socioeconomic status, etc.

To sum up, the present study investigated whether traditional Greek dance training would induce neuroplasticity effects on senior citizens. It employed network neuroscience in order to investigate the beneficial role of dancing on both global and local cortical level. To the best of our knowledge, it is the first attempt that demonstrated improved functional performance on cortical level through increased small world-property. This finding was mainly attributed to faster information flow and more accurate information integration among distant cortical regions (smaller characteristic path length). Neuroplasticity was more evident on specific modules such as the fronto-parietal and executive resting state networks known for being responsible for attention, motor planning and execution. The present computational approach seems to provide an integrative framework for evaluating the intervention impact on groups of senior citizens and to be robust for quantifying neuroplasticity effects on multi-level aspects of the functional cortical networks.

## Author Contributions

VZ designed and implemented the dance program, collected the data and prepared the initial draft of the manuscript. CF collected and analyzed the EEG data, guided the analysis, wrote EEG methodological parts, discussed the results and revised the manuscript. ER conceived the analysis of data. EP revised the manuscript. SD guided the study. PB co-guided the study and revised the manuscript.

## Conflict of Interest Statement

The authors declare that the research was conducted in the absence of any commercial or financial relationships that could be construed as a potential conflict of interest.

## Footnotes

^1^www.llmcare.gr
